# Treadmill Exercise Improves Fracture Toughness and Indentation Modulus without Altering the Nanoscale Morphology of Collagen in Mice

**DOI:** 10.1371/journal.pone.0163273

**Published:** 2016-09-21

**Authors:** Max A. Hammond, Tyler J. Laine, Alycia G. Berman, Joseph M. Wallace

**Affiliations:** 1 Weldon School of Biomedical Engineering, Purdue University, West Lafayette, IN, United States of America; 2 Department of Biomedical Engineering, Indiana University–Purdue University Indianapolis, Indianapolis, IN, United States of America; 3 Department of Orthopaedic Surgery, Indiana University School of Medicine, Indianapolis, IN, United States of America; University of Notre Dame, UNITED STATES

## Abstract

The specifics of how the nanoscale properties of collagen (e.g., the crosslinking profile) affect the mechanical integrity of bone at larger length scales is poorly understood despite growing evidence that collagen’s nanoscale properties are altered with disease. Additionally, mass independent increases in postyield displacement due to exercise suggest loading-induced improvements in bone quality associated with collagen. To test whether disease-induced reductions in bone quality driven by alterations in collagen can be rescued or prevented via exercise-mediated changes to collagen’s nanoscale morphology and mechanical properties, the effects of treadmill exercise and β-aminopropionitrile treatment were investigated. Eight week old female C57BL/6 mice were given a daily subcutaneous injection of either 164 mg/kg β-aminopropionitrile or phosphate buffered saline while experiencing either normal cage activity or 30 min of treadmill exercise for 21 consecutive days. Despite differences in D-spacing distribution (*P* = 0.003) and increased cortical area (tibial: *P* = 0.005 and femoral: *P* = 0.015) due to β-aminopropionitrile treatment, an overt mechanical disease state was not achieved as there were no differences in fracture toughness or 4 point bending due to β-aminopropionitrile treatment. While exercise did not alter (*P* = 0.058) the D-spacing distribution of collagen or prevent (*P* < 0.001) the β-aminopropionitrile-induced changes present in the unexercised animals, there were differential effects in the distribution of the reduced elastic modulus due to exercise between control and β-aminopropionitrile-treated animals (*P* < 0.001). Fracture toughness was increased (*P* = 0.043) as a main effect of exercise, but no significant differences due to exercise were observed using 4 point bending. Future studies should examine the potential for sex specific differences in the dose of β-aminopropionitrile required to induce mechanical effects in mice and the contributions of other nanoscale aspects of bone (e.g., the mineral–collagen interface) to elucidate the mechanism for the exercise-based improvements in fracture toughness observed here and the increased postyield deformation observed in other studies.

## Introduction

Type I collagen is the most abundant form of the most abundant protein in the human body, yet how its properties at the nanoscale influence the mechanical integrity of bone at larger length scales is poorly understood. There is growing evidence that the profile of collagen crosslinking plays an important role in determining the mechanical properties of bone, and this profile may be altered with disease [1]. Experimental osteolathyrism can be used to bridge this knowledge gap because it offers the ability to create a specific nanoscale deficiency in enzymatic collagen crosslinking. The disease is due to dietary intake of the β-aminopropionitrile (BAPN) toxin [2] and is well represented by animal models which are given a controlled dose of BAPN [3–5]. BAPN irreversibly blocks the action of lysyl oxidase (LOX), which inhibits the formation of enzymatic crosslinks [6]. Unlike non-enzymatic crosslinks, which can detrimentally affect bone, enzymatic crosslinks are typically associated with increased mechanical integrity.

Mechanical stimulation improves bone strength with increases in mass, but the effect of loading on bone quality due to alterations in the nanoscale morphology of collagen is unclear. Fracture risk varies among patients with the same bone mass due to variations in bone quality [7], and changes to the fundamental constituents of bone (e.g., collagen) could explain this variation. Post-yield mechanical properties are principally attributed to the quality of the organic matrix [8], and the increased postyield behavior in the bones of exercised mice may be driven by alterations to collagen [9]. In a previous study, treadmill exercise prevented BAPN-induced changes to the nanoscale morphology of collagen [10], but the effect of this protection on mechanical properties throughout bone’s hierarchical structure was not explored.

It was hypothesized that disease-induced reductions in bone quality driven by alterations in collagen can be rescued or prevented via exercise-mediated changes to collagen’s nanoscale morphology and mechanical properties, restoring tissue and structural mechanical integrity to control levels. To test this hypothesis, changes in the morphology, composition, and mechanical properties of bone from a murine model of osteolathyrism under sedentary and exercise conditions were investigated at several relevant length scales throughout bone’s hierarchical structure. Efforts to link nanoscale differences to tissue and structural level mechanical properties have lacked multiscale mechanical information that fully extends to nanoscale mechanical differences, and these data will be critical to uncovering the specific causative factors that drive decreased bone mechanical function. The long term goal of this research is to use an exercise program for the treatment and prevention of collagen-based bone disease by altering collagen at the nanoscale.

## Materials and Methods

### Animals

Seven week old female C57BL/6 mice were given 1 week to acclimate to the animal facility and were separated into 4 weight-matched groups (*n* = 15 per group) at 8 weeks of age with prior approval from the Indiana University–Purdue University Indianapolis School of Science Institutional Animal Care and Use Committee (Protocol #SC210R). Mice were subjected to either normal cage activity (Sed) or an exercise regimen (Ex) previously shown to alter bone structural and tissue-level mechanical properties [11]. All mice had access to food, water, and cage activity ad libitum. Ex animals ran on a treadmill (Animal Treadmill: Exer 3/6, Columbus Instruments, Columbus, OH) for 30 min/day at 12 m/min on a 5° incline for 21 consecutive days. The mice received daily 200 μL subcutaneous injections of either phosphate buffered saline (PBS; Gibco PBS pH 7.4, Thermo Fisher Scientific, Waltham, MA) or a 164 mg/kg dose of BAPN (300 mg/kg BAPN fumarate; Sigma-Aldrich, St. Louis, MO) in PBS, previously shown to alter the nanoscale morphology of collagen in this model [10]. Mice were weighed every other day beginning on day 0 and ending on day 20 to ensure proper dosage of BAPN. A 100 μL injection of 30 mg/kg calcein (Sigma) was given subcutaneously on days 13 and 20 (9 and 2 days before sacrifice) for dynamic histomorphometric assessment of bone formation. After sacrifice by CO_2_ inhalation at 11 weeks of age, left and right femora and tibiae were removed, stripped of soft tissue, wrapped in PBS-soaked gauze, and stored at −20°C until needed.

### Atomic force microscopy

To prepare the right femur for atomic force microscopy (AFM), a section of the mid-diaphysis, beginning from the widest point of the third trochanter and extending 4.00 mm distally, was sectioned using a low-speed sectioning saw (IsoMet Low Speed Saw, Buehler, Lake Bluff, IL) and mounted with the posteromedial side up to a steel disk using cyanoacrylate glue (Loctite Super Glue ULTRA Gel Control, Loctite, Westlake, Ohio). The section was polished with a 3 μm diamond suspension (MetaDi 3 μm, Buehler), sonicated for 1 min in ultrapure water, polished with a 0.05 μm alumina powder (MicroPolish II, Buehler), and sonicated for an additional minute in ultrapure water (*n* = 10 per group). Beginning 500 μm from the distal end of the section, 25 × 25 μm images were acquired from 3 locations spaced 250 μm apart on the posterior side of the polished region using a BioScope Catalyst AFM (Bruker, Santa Barbara, CA) with a tungsten carbide coated probe which had a nominal tip radius of 20 nm (HSC-20-125C650-MC-R, Nanoscience Instruments, Phoenix, AZ) in peak force tapping mode while submerged in ultrapure water (*n* = 10 per group). Approximately 144 indentations spaced at least 2 μm apart to a trigger force of 1.5 μN at a speed of 0.5 Hz were performed at each location. Using a custom written MATLAB (Mathworks, Natick, MA) script, a linear baseline was fit from 0% to 90% of the approach and retraction curves and the reduced elastic modulus (*E*_r_) was calculated from the retraction curve in a region spanning 25% to 75% of the maximum force using the Sneddon method [12, 13]. Only indents with acceptable levels of noise, points in the retraction curve, and fit to the Sneddon model were used for subsequent statistical tests resulting in a total of 100 to 200 indents per sample. A single probe was used for all indentations and all samples. A micrometer was used for the sections, and the AFM stage allowed for precise distances from the edge of the section and the polished regions to ensure each location between samples was of similar tissue age. The posteromedial surface was selected because the most bone was formed in this region over the course of the study so that the locations chosen would interrogate tissue formed during the BAPN treatment. Following indentation, each section was wrapped in PBS-soaked gauze and stored at −20°C until needed.

In order to assess collagen morphology, each section was thawed at room temperature and underwent 3 cycles of a 14 min 0.5 M ethylenediaminetetraacetic acid (Invitrogen UltraPure EDTA, Life Technologies, Carlsbad, CA) treatment at a pH of 8.0 followed by sonication for 5 min in ultrapure water (*n* = 10 per group). From 3 to 4 locations in approximately the same region previously used for indentation, 3.5 × 3.5 μm images were acquired using a BioScope Catalyst AFM with a silicon probe which had a nominal tip radius of 2 nm (ScanAsyst-Fluid+, Bruker). At each location, 15 to 20 fibrils were analyzed in error images (approximately 50 fibrils per bone) as previously described [10, 14–18]. Briefly, 2D fast Fourier transforms were performed on an area of interest over individual fibrils so that the first harmonic peak from the power spectrum represented the D-spacing for that fibril.

### Raman spectroscopy

After being thawed at room temperature, right tibiae were stripped of their periosteum by lightly scraping with a scalpel. To keep the bones fully hydrated during imaging, each sample was placed in an ultrapure water bath with only the anterior surface exposed to air. Raman spectroscopy was performed with a LabRAM HR 800 Raman Spectrometer (HORIBA Jobin Yvon, Edison, NJ) with an integrated confocal BX41 microscope (Olympus, Tokyo, Japan). Spectra were acquired from 4 to 5 locations per sample approximately 1 mm apart along the anterior surface of the bone, distal to the tibiofibular junction (TFJ; *n* = 14 to 15 per group). A 660 nm laser was focused to a spot size of approximately 10 μm using a 50× objective (NA = 0.75) and five 20 s acquisitions were acquired through a 200 μm confocal hole at each location to restrict the Raman signal to the outer presumably young tissue formed under the influence of BAPN. Spectra were baseline corrected using asymmetric least squares smoothing and denoised using Daubechies 10 wavelet in OriginPro 2016 (OriginLab, Northampton, MA). OriginPro was used to integrate the PO_4_^3−^ *v*1, CO_3_^2−^ *v*1, Amide III, CH_2_ wag, and the Amide I bands as previously described [18]. Relative levels of type B carbonate substitution were found by the band area ratio of CO_3_^2−^ *v*1 / PO_4_^3−^ *v*1. The band area ratios PO_4_^3−^ *v*1 / Amide I, PO_4_^3−^ *v*1 / CH_2_ wag, and PO_4_^3−^ *v*1 / Amide III were calculated as metrics of matrix mineralization. In OriginPro, a single Gaussian function was fit to the PO_4_^3−^ *v*1 peak and the crystallinity/maturity was determined by the inverse of the full width at half maxima of the fitted peak. Each sample was wrapped in PBS-soaked gauze and stored at −20°C until needed.

### Dynamic histomorphometry

After Raman, the right tibiae were dehydrated in a series of graded ethanol solutions (70%, 80%, 90%, 95%, and 100%), cleared in xylenes (Sigma-Aldrich), infiltrated with unpolymerized methyl methacrylate (Sigma-Aldrich) containing 4% dibutyl phthalate (Sigma-Aldrich), and embedded in poly(methyl methacrylate) using additional methyl methacrylate and 0.8% Perkadox 16 catalyst (AkzoNobel, Amsterdam, Netherlands). Beginning 1 mm proximal to the TFJ, 3 serial sections approximately 120 μm thick were cut using a 3241 Diamond Wire Saw (Delaware Diamond Knives, Wilmington, DE), briefly cleared in xylenes, mounted to glass slides using Eukitt (Sigma-Aldrich), and ground to a final thickness of approximately 20 μm using 600 grit silicon carbide sandpaper (LECO, St. Joseph, MI; *n* = 14 to 15 per group). A Leica DM 3000 photomicroscope (Leica Microsystems, Buffalo Grove, IL) with integrated BIOQUANT OSTEO 2012 software (BIOQUANT Image Analysis Corporation, Nashville, TN) was used to measure the perimeter (B.Pm), single label perimeter, double label perimeter, and the interlabel width on the periosteal and endocortical surfaces for 2 sections per bone. These measurements were used to calculate the periosteal mineralizing perimeter (Ps.M.Pm/B.Pm), endocortical mineralizing perimeter (Ec.M.Pm/B.Pm), periosteal mineral apposition rate (Ps.MAR), endocortical mineral apposition rate (Ec.MAR), periosteal bone formation rate (Ps.BFR/B.Pm), and endocortical bone formation rate (Ec.BFR/B.Pm) for each surface using standard methods and nomenclature [19].

### Micro-computed tomography

Left femora and tibiae were thawed at room temperature prior to micro-computed tomography (μCT). Samples were wrapped in Parafilm M (Bemis, Oshkosh, WI) to maintain hydration and were scanned in air with the long axis of the bone vertically oriented using a Skyscan 1172 μCT system (Bruker microCT, Kontich, Belgium; *n* = 15 per group). Scans were performed with a source voltage of 59 kV and current of 167 μA through a 0.5 mm Al filter. An isotropic 11.9 μm voxel size was used for femoral scans and a 17.2 μm voxel size was used for tibial scans in order to capture the anatomical locations necessary for defining the cortical standard sites and for properly aligning the bones. NRecon (Bruker microCT) was used to reconstruct voxels with attenuation coefficients ranging from 0 to 0.11 mm^−1^, apply a beam hardening correction of 40%, and apply a ring artifact correction of 5. Mineral density was calculated using daily scans of manufacturer supplied hydroxyapatite (HA) phantoms of 0.25 and 0.75 g/cm^3^. Reconstructed scans were rotated using Dataviewer (Bruker microCT) to ensure precise vertical alignment. For the femora, the metaphyseal region of interest extended 2 mm proximally from the proximal end of the distal growth plate. For the tibiae, the metaphyseal region of interest extended 1.7 mm distally from the distal end of the proximal growth plate. A custom MATLAB script segmented the cortical shell from the trabecular region of interest which followed the contour of the cortical shell in each slice. CTAn (Bruker microCT) was used to compute bone volume fraction (BV/TV), trabecular thickness (Tb.Th), trabecular separation (Tb.Sp), trabecular number (Tb.N), structure model index (SMI), connectivity density (Conn.Dn), and volumetric bone mineral density (vBMD). The cortical standard site was defined as a 7 slice region centered on the slice that was 75% the distance between the distal growth plate and the widest point of the third trochanter for the femora and 50% the total length for the tibiae. A custom MATLAB script was used to calculate total area (Tt.Ar), marrow area (Ma.Ar), cortical area (Ct.Ar), cortical width (Ct.Wi), width of the anteroposterior axis (AP.Wi), width of the mediolateral axis (ML.Wi), AP.Wi to ML.Wi ratio (AP.Wi/ML.Wi), periosteal perimeter (Ps.Pm), endocortical perimeter (Ec.Pm), section modulus (*S*), and tissue mineral density (TMD). Following imaging, samples were wrapped in PBS-soaked gauze and stored at −20°C.

### Four point bending

After μCT, the left tibiae were thawed at room temperature and monotonically tested to failure using 4 point bending with a loading span of 3 mm and a support span of 9 mm in displacement control at a rate of 0.025 mm/s while submerged in PBS (*n* = 13 to 15 per group). The TFJ was positioned just outside of the loading span and the bone was tested in the mediolateral direction with the medial surface in tension. The distance from the distal end of the tibia to the initiation of failure on the medial surface was measured with calipers. The second moment of inertia about the anteroposterior axis and the extreme fiber in tension were obtained from the μCT images using a seven slice region centered on the failure site and were used to map load-displacement to stress-strain curves. Pre- and postyield mechanical properties were calculated with a custom written MATLAB script as previously described [20].

### Fracture toughness

A notch was cut into the medullary cavity through the anteromedial side of the left femur at the mid-diaphysis using a scalpel blade lubricated with a 1 μm diamond suspension (MetaDi 1 μm, Buehler) to a depth not exceeding the midpoint of the section (*n* = 12 to 15 per group). The samples were tested to failure in 3 point bending with a support span of 8 mm at a rate of 1 μm/s and the notch in tension directly under the applied load. The location of the fracture site was measured with calipers and the geometric properties of the fracture site were determined using μCT data. After dehydration with a series of graded ethanol (70% to 100% as before), samples were mounted to a stainless steel stub using carbon tape (Electron Microscopy Sciences, Hatfield, PA), gold-coated, and the fracture surface was imaged using a scanning electron microscope (JSM-7800F, JEOL, Peabody, MA) to obtain the angles of stable and unstable crack growth. The force and displacement data, geometric properties, and crack growth angles were used to calculate fracture stress intensity factor (*K*_c_) following a linear elastic fracture mechanics approach using a custom MATLAB script according to the fracture instability method [21, 22].

### Statistical analysis

Multiple locations within a single sample were averaged to produce the sample value used in mean comparisons. A two-way ANOVA was used to test the main effects of BAPN treatment, exercise, and their interaction. If a significant interaction was present, a one-way model was substituted with a post hoc Tukey test if needed. Violations of normality or homoscedasticity were determined with a Shapiro–Wilk test or a Brown–Forsythe test, respectively (*P* < 0.05). These violations were overcome with an appropriate transformation where necessary. To compare the D-spacing and *E*_r_ distributions, a one-way Anderson–Darling test (AD tests) was used with post hoc pairwise comparisons to test for different cumulative distribution functions between groups. A Bonferroni correction was used for these pairwise comparisons which lowered the threshold for a comparison to be considered significant to *P* < 0.0083. Custom MATLAB scripts were used to perform the ANOVA, check assumptions, apply transformations, and perform the AD tests. All data are presented as mean ± standard deviation (SD). Each assay and analysis was preformed in randomized order by a single operator while blinded.

## Results

### Animals

At the initiation of exercise, the grand mean body weight for all groups was 18.1 g (Sed PBS 18.0 ± 0.6 g, Sed BAPN 18.1 ± 0.8 g, Ex PBS 18.1 ± 0.7 g, and Ex BAPN 18.3 ± 0.9 g) which was within the expected body weight for the age, strain, and sex of mice used [23]. No animal had difficulty completing the exercise protocol. Body weights did not differ at any time throughout the study. All groups gained between 12% and 15% body mass over the 3 week exercise period (Sed PBS 20.8 ± 0.9 g, Sed BAPN 20.4 ± 0.9 g, Ex PBS 20.3 ± 1.4 g, and Ex BAPN 20.8 ± 1.0 g). After accounting for animal to animal variability and growth during the study, the average delivered dose of BAPN throughout the study for both BAPN groups was 159 ± 11 mg/(kg·day).

### Nanoscale indentation

**Fig 1:**
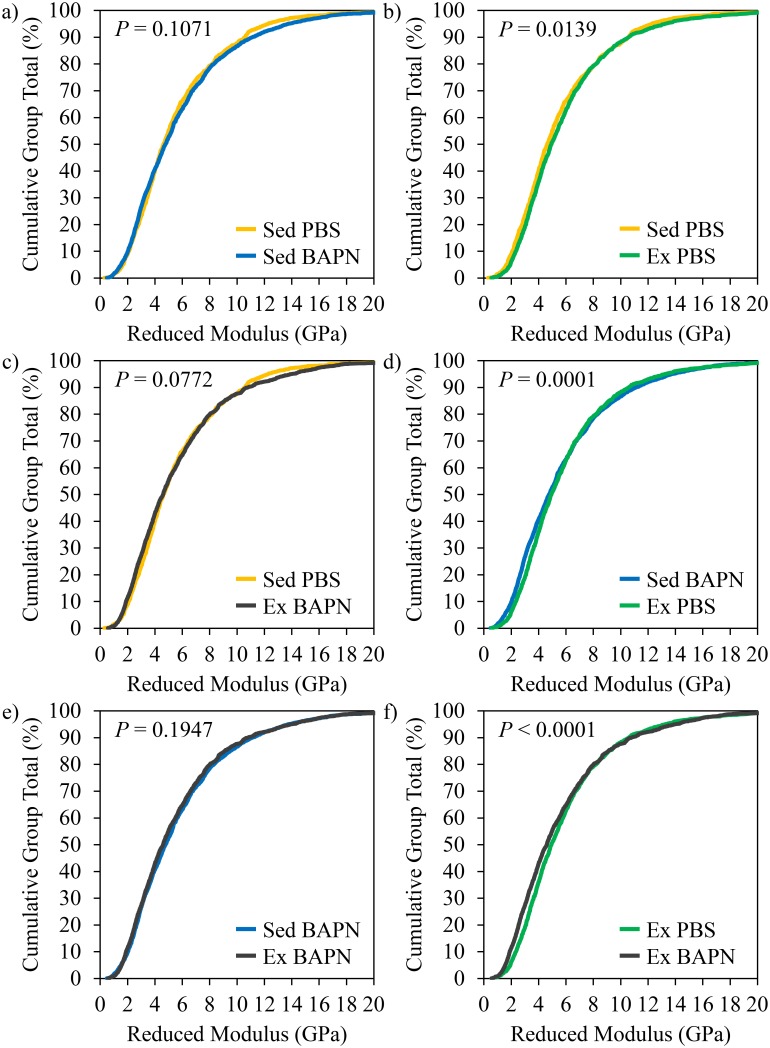
*E*_r_ distributions. Pairwise comparisons of *E*_r_ from nanoscale indentation. a) Sed BAPN vs. Sed PBS. b) Ex PBS vs. Sed PBS. c) Ex BAPN vs. Sed PBS. d) Ex PBS vs. Sed BAPN. e) Ex BAPN vs. Sed BAPN. f) Ex BAPN vs. Ex PBS.

The average *E*_r_ for each group was 5576 ± 765 MPa for Sed PBS (*n* = 10, 1069 indents), 6099 ± 1793 MPa for Sed BAPN (*n* = 10, 2294 indents), 5986 ± 1254 MPa for Ex PBS (*n* = 10, 1337 indents), and 5903 ± 2013 MPa for Ex BAPN (*n* = 10, 1698 indents). There were no significant mean differences for the main effects of BAPN, Ex, or their interaction. However, there were differences in the distribution of *E*_r_ (AD test *P* < 0.001, Fig 1). Post hoc pairwise comparisons revealed differences for Sed BAPN vs. Ex PBS (*P* = 0.0001) and Ex PBS vs. Ex BAPN (*P* < 0.0001). While there were no other significant pairwise comparisons, there was a non-significant trend for an upward shift in Ex PBS vs. Sed PBS (*P* = 0.0139) that would have reached significance if it had not been for the conservative Bonferroni correction.

### Collagen morphology

**Fig 2:**
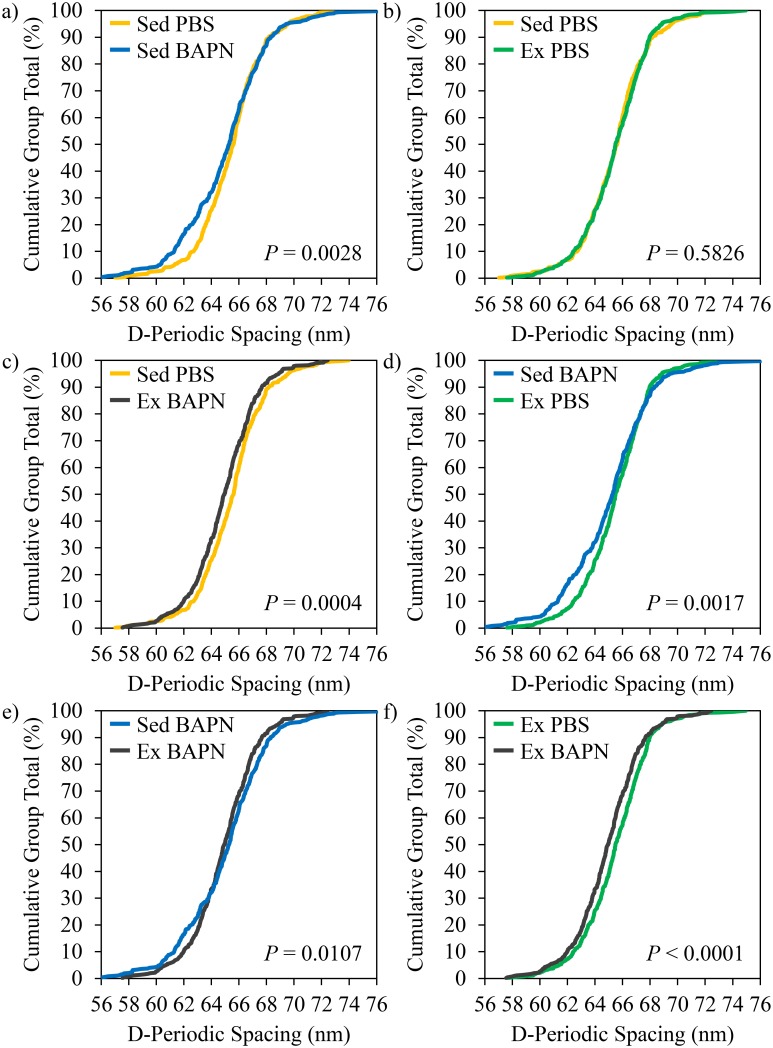
D-spacing distributions. Pairwise comparisons of D-spacing from AFM. a) Sed BAPN vs. Sed PBS. b) Ex PBS vs. Sed PBS. c) Ex BAPN vs. Sed PBS. d) Ex PBS vs. Sed BAPN. e) Ex BAPN vs. Sed BAPN. f) Ex BAPN vs. Ex PBS.

The average D-spacing for each group was 65.4 ± 0.7 nm for Sed PBS (*n* = 10, 492 fibrils), 65.1 ± 1.2 nm for Sed BAPN (*n* = 10, 479 fibrils), 65.5 ± 0.9 nm for Ex PBS (*n* = 10, 507 fibrils), and 64.9 ± 0.9 nm for Ex BAPN (*n* = 10, 513 fibrils). There were no significant mean differences for the main effects of BAPN, Ex, or their interaction. However, there were differences in D-spacing distribution (AD test *P* < 0.001, Fig 2). BAPN treatment altered D-spacing distribution in both Sed and Ex conditions (Sed PBS vs. Sed BAPN *P* = 0.0028 and Ex PBS vs. Ex BAPN *P* < 0.0001). Concurrent exercise with BAPN treatment did not prevent a BAPN-induced shift in D-spacing (Sed PBS vs. Ex BAPN *P* = 0.0004) as was noted in previous work [10], and exercise alone did not have a significant effect (Fig 2b). BAPN’s effect was not statistically different between Sed and Ex conditions after employing the Bonferroni correction (Sed BAPN vs. Ex BAPN *P* = 0.0107) and Sed BAPN was significantly different from Ex PBS (*P* = 0.0017).

### Raman spectroscopy

One sample was excluded from the Sed BAPN group because the periosteum was insufficiently removed. All other groups had 15 samples. As seen in Table 1, there were no significant differences for any Raman parameter. There was a trend for reduced PO_4_^3—^ *v*1 / Amide I with BAPN and increased crystallinity/maturity with Ex but both failed to reach significance (*P* = 0.096 for both parameters). There were no significant interactive effects between BAPN and Ex.

**Table 1:** Raman spectroscopy on medial surface of distal right tibiae.

	Sed PBS	Sed BAPN	Ex PBS	Ex BAPN
Crystallinity/maturity	0.0526 ± 0.0008	0.0523 ± 0.0008	0.0528 ± 0.0006	0.0527 ± 0.0012
PO_4_^3−^ *v*1 / Amide I	1.55 ± 0.29	1.43 ± 0.31	1.51 ± 0.19	1.41 ± 0.31
CO_3_^2−^ *v*1 / PO_4_^3−^ *v*1	0.281 ± 0.046	0.285 ± 0.043	0.287 ± 0.032	0.318 ± 0.055
PO_4_^3−^ *v*1 / Amide III	2.63 ± 0.45	2.48 ± 0.36	2.57 ± 0.29	2.40 ± 0.45
PO_4_^3−^ *v*1 / CH_2_	3.18 ± 0.44	3.03 ± 0.32	3.03 ± 0.24	2.90 ± 0.40

Values presented as mean ± SD. Sed PBS *n* = 15. Sed BAPN *n* = 14. Ex PBS *n* = 15. Ex BAPN *n* = 15.

### Dynamic histomorphometry

One sample per group was excluded from Sed PBS, Sed BAPN, and Ex BAPN due to difficulties during processing or the presence of woven bone. The Ex PBS group had 15 samples. A one-way model was used for Ec.M.Pm/B.Pm and Ec.MAR due to significant interactive effects (*P* = 0.045 and *P* = 0.020, respectively). As seen in Table 2, Ec.MAR was significantly different between groups (*P* = 0.014) and post hoc tests revealed that the Sed PBS group had a larger Ec.MAR than the Sed BAPN and Ex PBS groups. Despite a significant interaction, the one-way ANOVA was not significant for Ec.M.Pm/B.Pm and no post hoc tests were performed. There was a non-significant trend for increased Ps.MAR with BAPN (*P* = 0.071).

**Table 2:** Dynamic histomorphometry of tibial mid-diaphysis.

	Sed PBS	Sed BAPN	Ex PBS	Ex BAPN
Ec.M.Pm/B.Pm (%) #	59.2 ± 9.9	66.9 ± 8.8	66.8 ± 12.3	63.5 ± 9.2
Ec.MAR (μm/day) #	1.19 ± 0.14	0.98 ± 0.18 ^a^	0.99 ± 0.16 ^a^	1.02 ± 0.24
Ec.BFR/B.Pm (μm^3^/μm^2^/year)	258 ± 57	244 ± 66	244 ± 68	236 ± 62
Ps.M.Pm/B.Pm (%)	71.4 ± 9.1	75.0 ± 7.9	76.5 ± 7.7	73.8 ± 7.7
Ps.MAR (μm/day)	1.34 ± 0.27	1.40 ± 0.28	1.21 ± 0.24	1.38 ± 0.21
Ps.BFR/B.Pm (μm^3^/μm^2^/year)	353 ± 105	387 ± 113	341 ± 90	373 ± 78

Values presented as mean ± SD. Sed PBS *n* = 14. Sed BAPN *n* = 14. Ex PBS *n* = 15. Ex BAPN *n* = 14.

^#^ significant interaction.

^a^ Different from Sed PBS.

### Femoral cortical analysis

As seen in Table 3, Ct.Ar (*P* = 0.015), Ct.Wi (*P* = 0.049), ML.Wi (*P* = 0.019), and *S* (*P* = 0.006) were increased due to BAPN. Although significant, these changes were modest. There were no significant Ex or interactive effects for any parameter.

**Table 3:** Cortical analysis of femoral standard site.

	Sed PBS	Sed BAPN	Ex PBS	Ex BAPN
Tt.Ar (mm^2^)	1.56 ± 0.05	1.60 ± 0.06	1.54 ± 0.06	1.57 ± 0.06
Ma.Ar (mm^2^)	0.826 ± 0.042	0.844 ± 0.046	0.820 ± 0.046	0.820 ± 0.036
Ct.Ar (mm^2^) *	0.734 ± 0.028	0.754 ± 0.029	0.724 ± 0.043	0.746 ± 0.028
Ct.Wi (μm) *	193 ± 7	196 ± 6	191 ± 11	196 ± 5
AP.Wi (mm)	1.24 ± 0.03	1.25 ± 0.04	1.23 ± 0.03	1.23 ± 0.04
ML.Wi (mm) *	1.64 ± 0.04	1.68 ± 0.04	1.64 ± 0.03	1.66 ± 0.04
AP.Wi / ML.Wi	0.755 ± 0.026	0.745 ± 0.032	0.747 ± 0.021	0.744 ± 0.030
Ps.Pm (mm)	5.27 ± 0.07	5.33 ± 0.10	5.25 ± 0.09	5.28 ± 0.09
Ec.Pm (mm)	3.98 ± 0.10	4.03 ± 0.11	3.97 ± 0.09	3.98 ± 0.10
*S* (mm^3^) *	0.229 ± 0.011	0.238 ± 0.013	0.223 ± 0.016	0.233 ± 0.013
TMD (g/cm^3^ HA)	1.511 ± 0.022	1.509 ± 0.030	1.519 ± 0.024	1.524 ± 0.023

Values presented as mean ± SD.

* main effect of BAPN.

### Tibial cortical analysis

A similar cortical expansion due to BAPN was observed in the tibia, but the effects were stronger in the tibia than they were in the femur (Table 4). In addition to Ct.Ar (*P* = 0.005), Ct.Wi (*P* = 0.018), ML.Wi (*P* = 0.041), and *S* (*P* = 0.012), Tt.Ar (*P* = 0.003), AP.Wi (*P* = 0.008), Ps.Pm (*P* = 0.012), and Ec.Pm (*P* = 0.018) were significantly increased due to BAPN treatment (Table 4). TMD was significantly reduced due to Ex and was the only cortical parameter where Ex had an effect (Table 4). There were non-significant trends for decreased AP.Wi (*P* = 0.096) and decreased AP.Wi/ML.Wi (*P* = 0.064) with Ex, and there were neither significant effects of BAPN nor significant interactive effects for these parameters.

**Table 4:** Cortical analysis of tibial standard site.

	Sed PBS	Sed BAPN	Ex PBS	Ex BAPN
Tt.Ar (mm^2^)	1.04 ± 0.04	1.09 ± 0.07	1.04 ± 0.06	1.08 ± 0.07
Ma.Ar (mm^2^)	0.350 ± 0.022	0.362 ± 0.024	0.347 ± 0.020	0.354 ± 0.027
Ct.Ar (mm^2^) *	0.695 ± 0.042	0.730 ± 0.052	0.692 ± 0.049	0.730 ± 0.047
Ct.Wi (μm) *	241 ± 13	248 ± 13	241 ± 14	251 ± 11
AP.Wi (mm)	1.23 ± 0.06	1.25 ± 0.05	1.20 ± 0.04	1.24 ± 0.04
ML.Wi (mm) *	1.17 ± 0.03	1.21 ± 0.06	1.18 ± 0.04	1.20 ± 0.06
AP.Wi / ML.Wi	1.052 ± 0.052	1.041 ± 0.041	1.017 ± 0.034	1.034 ± 0.050
Ps.Pm (mm)	4.57 ± 0.15	4.68 ± 0.20	4.53 ± 0.12	4.63 ± 0.15
Ec.Pm (mm)	2.66 ± 0.09	2.73 ± 0.10	2.65 ± 0.08	2.69 ± 0.10
*S* (mm^3^) *	0.125 ± 0.009	0.135 ± 0.013	0.127 ± 0.011	0.133 ± 0.013
TMD (g/cm^3^ HA) †	1.160 ± 0.044	1.207 ± 0.077	1.151 ± 0.069	1.158 ± 0.070

Values presented as mean ± SD.

* main effect of BAPN.

^†^ main effect of Ex.

### Femoral trabecular analysis

Whereas BAPN treatment dominated the effects on cortical geometry, the opposite was true in the trabecular compartment. As seen in Table 5, Ex increased BV/TV (*P* = 0.014), decreased Tb.Sp (*P* = 0.011), increased Tb.N (*P* = 0.018), decreased SMI (*P* = 0.013), and increased vBMD (*P* = 0.001). There was a non-significant trend for increased Conn.Dn due to Ex (*P* = 0.062). There were no significant interactive effects.

**Table 5:** Trabecular analysis of distal femora.

	Sed PBS	Sed BAPN	Ex PBS	Ex BAPN
BV/TV (%) †	10.4 ± 1.4	10.4 ± 1.0	11.0 ± 1.3	11.5 ± 1.4
Tb.Th (μm)	69.9 ± 2.4	69.9 ± 2.2	69.3 ± 1.8	70.6 ± 2.0
Tb.Sp (μm) †	278 ± 25	275 ± 19	265 ± 16	262 ± 15
Tb.N (1/mm) †	1.49 ± 0.23	1.49 ± 0.12	1.59 ± 0.17	1.63 ± 0.19
SMI †	2.45 ± 0.12	2.47 ± 0.08	2.41 ± 0.08	2.38 ± 0.12
Conn.Dn (1/mm^3^)	102 ± 18	99 ± 10	108 ± 13	107 ± 12
vBMD (g/cm^3^ HA) †	0.146 ± 0.016	0.148 ± 0.012	0.156 ± 0.018	0.165 ± 0.013

Values presented as mean ± SD.

^†^ main effect of Ex.

### Tibial trabecular analysis

As seen in Table 6, Ex increased BV/TV (*P* = 0.002), Tb.Th (*P* = 0.014), Tb.N (*P* = 0.004), and vBMD (*P* = 0.009). There was a non-significant trend for decreased SMI due to exercise (*P* = 0.074). BAPN significantly decreased SMI (*P* = 0.023) and increased Conn.Dn (*P* = 0.048). There was a non-significant trend for increased BV/TV due to BAPN (*P* = 0.099). There were no significant interactive effects.

**Table 6:** Trabecular analysis of proximal tibiae.

	Sed PBS	Sed BAPN	Ex PBS	Ex BAPN
BV/TV (%) †	12.0 ± 2.2	12.1 ± 1.4	12.9 ± 1.8	14.2 ± 1.8
Tb.Th (μm) †	81.3 ± 3.5	81.8 ± 2.7	82.9 ± 2.3	84.1 ± 3.1
Tb.Sp (μm)	366 ± 41	375 ± 22	360 ± 25	356 ± 25
Tb.N (1/mm) †	1.47 ± 0.23	1.48 ± 0.14	1.55 ± 0.19	1.69 ± 0.19
SMI *	2.25 ± 0.11	2.21 ± 0.12	2.23 ± 0.11	2.13 ± 0.12
Conn.Dn (1/mm^3^) *	65.1 ± 13.6	70.8 ± 11.9	66.3 ± 12.9	73.9 ± 12.3
vBMD (g/cm^3^ HA) †	0.141 ± 0.023	0.144 ± 0.022	0.152 ± 0.013	0.159 ± 0.011

Values presented as mean ± SD.

* main effect of BAPN.

^†^ main effect of Ex.

### Four point bending

One sample was excluded from the Sed PBS group and 2 samples each were excluded from Sed BAPN and Ex BAPN due to errors positioning the bone during testing. As seen in Table 7, there were no significant main effects for any parameter. There was a non-significant increase in ultimate force due to BAPN (*P* = 0.070). There were no significant interactive effects for any parameter.

**Table 7:** Structure- and tissue-level mechanical properties from 4 point bending of left tibiae.

	Sed PBS	Sed BAPN	Ex PBS	Ex BAPN
Yield Force (N)	13.1 ± 1.3	14.0 ± 1.3	13.3 ± 2.8	13.7 ± 1.2
Ultimate Force (N)	15.2 ± 1.3	16.3 ± 2.0	15.4 ± 2.8	16.2 ± 1.1
Displacement to Yield (μm)	233 ± 14	240 ± 65	230 ± 32	233 ± 28
Postyield Displacement (μm)	533 ± 304	593 ± 288	504 ± 275	476 ± 169
Total Displacment (μm)	766 ± 306	834 ± 264	734 ± 275	709 ± 178
Stiffness (N/mm)	64.1 ± 6.5	69.5 ± 14.8	67.6 ± 15.5	67.3 ± 8.7
Work to Yield (mJ)	1.66 ± 0.20	1.79 ± 0.35	1.66 ± 0.44	1.73 ± 0.29
Postyield Work (mJ)	6.30 ± 3.06	7.89 ± 4.10	6.44 ± 3.60	6.04 ± 1.72
Total Work (mJ)	7.96 ± 3.07	9.68 ± 3.94	8.10 ± 3.81	7.77 ± 1.75
Yield Stress (MPa)	184 ± 33	183 ± 30	173 ± 53	179 ± 38
Ultimate Stress (MPa)	213 ± 37	214 ± 37	199 ± 54	210 ± 38
Strain to Yield (mε)	18.3 ± 1.7	19.4 ± 6.5	18.4 ± 2.1	18.5 ± 2.0
Total Strain (mε)	59.2 ± 21.6	68.4 ± 29.1	58.3 ± 21.4	56.8 ± 14.7
Modulus (GPa)	11.6 ± 3.0	11.7 ± 3.7	11.0 ± 3.9	11.1 ± 2.6
Resilience (MPa)	1.81 ± 0.26	1.87 ± 0.40	1.70 ± 0.50	1.80 ± 0.41
Toughness (MPa)	8.83 ± 3.91	9.94 ± 3.70	8.24 ± 3.91	8.01 ± 2.03

Values presented as mean ± SD.

### Fracture toughness

**Fig 3:**
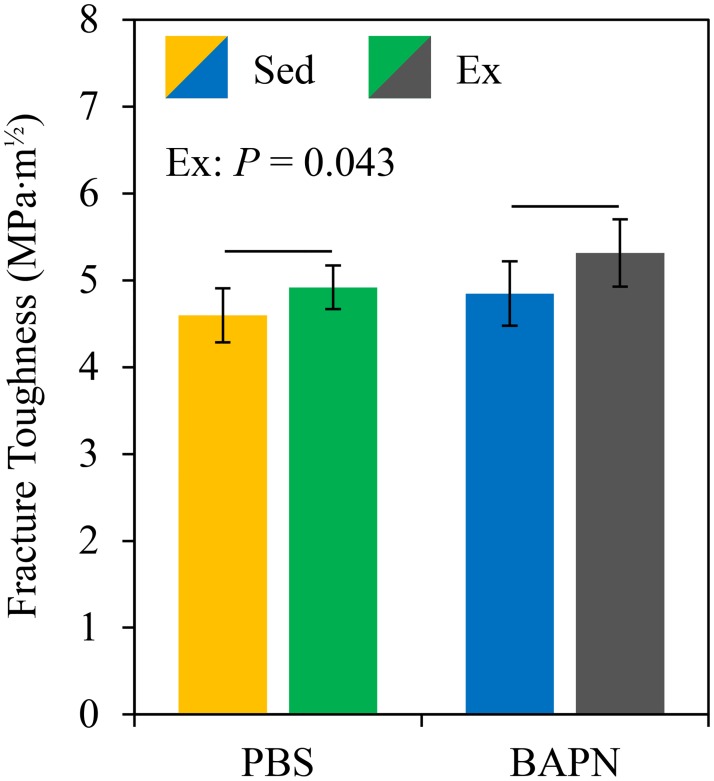
Exercise increased fracture toughness. Exercise improved the fracture instability *K*_c_ for PBS and BAPN treated mice.

Three samples were excluded from the Ex BAPN group and 1 sample was excluded from the Ex PBS group because a clear transition to unstable crack growth could not be identified. As seen in Fig 3, Ex significantly increased *K*_c_ (*P* = 0.043). There were no significant interactive effects.

## Discussion

The goal of this study was to assess the effects of BAPN treatment and treadmill exercise on the morphology, composition, and mechanics of bone. The ability of exercise to prevent or compensate for disease-induced reductions in mechanical integrity was evaluated at several relevant length scales. While composition was not altered due to either BAPN or exercise, BAPN had effects on cortical structure and nanoscale morphology, and exercise affected trabecular microarchitecture and tissue-level mechanics.

Positively skewed data is typical of AFM indentation measures of modulus [13, 24, 25], and this large variance often masks differences between groups when only comparisons between group means are made. Comparisons between group distributions using AD tests are useful because they are not heavily influenced by outliers at the extreme ends of the distribution, which allow for differences driven by the majority of the population to be detected. Exercise did not affect the *E*_r_ of the mineralized bone matrix in BAPN treated animals (Fig 1e). While neither the Sed BAPN nor the Ex BAPN group was significantly different from Sed PBS (Fig 1a and 1b), both distributions hugged the lower end of Sed PBS’s distribution. When comparing these groups to Ex PBS, an effect of exercise that did not reach significance when comparing Sed PBS to Ex PBS (Fig 1c) was revealed (Fig 1d and 1f) due to the presence of slightly lower values in BAPN groups. While the comparison between Sed PBS and Ex PBS did not reach significance due to the conservative Bonferroni correction, the *P* value was low enough to be of interest as a possible non-significant trend (*P* < 0.0167) for future investigation. Therefore, exercise shifts the indentation modulus to higher values, but the presence of BAPN silences this effect. The prevention of exercise-induced improvements in *E*_r_ by BAPN treatment further supports the idea that exercise-mediated improvements in bone quality are primarily collagen-driven [8, 26].

The downward shift in collagen D-spacing distribution due to BAPN (Fig 2a) was similar to previous reports [10], further indicating that BAPN-induced suppression of enzymatic crosslinks alters collagen morphology. Although indentation suggests exercise-induced improvements are collagen-mediated, no effect on the D-spacing distribution was observed with exercise which again agrees with the previous study [10]. While D-spacing distribution is a robust parameter and is altered due to wide variety of disease states in bone and tendon [10, 13–15, 18, 21, 25, 27, 28], D-spacing analysis in bone is performed on demineralized tissues. Together, the lack of D-spacing changes and mechanical evidence that exercise-induced improvements are driven by matrix-mediated effects [8, 26] suggest the mechanism behind these improvements is related to the collagen–mineral interface or possibly noncollagneous proteins (NCPs) rather than direct changes in collagen morphology. Future studies should examine the effect of exercise on the collagen–mineral interface and dilatational bands (as a measure of NCP-mediated effects) to explain the exercise-induced increases in fracture toughness noted here [29].

The ability of exercise to prevent BAPN-induced changes in collagen morphology, as noted in the previous study [10], was not observed in the present study. While the same age, sex, and strain of mouse and the same exercise protocol were used in this study, AFM analysis was performed on the posterolateral surface in the current study whereas the anterior surface was used previously [10]. The posterolateral surface was selected because the primary direction of cortical drift in the femur is in the posterolateral direction [30]. Therefore, the largest volume effected by BAPN was expected to occur near the posterolateral surface due to the presence of primarily new tissue that would have been exposed to BAPN during its formation. Because the current study used the opposite surface of the bone, the local stress environment was reversed (tension vs. compression). Additionally, the magnitude of stress experienced on the posterolateral surface was presumably lower here due to a 8.5% greater section modulus in the current study (Table 3) than in the previous study. The mechanism for preventing the downward shift in D-spacing shifts was suggested to involve exercise-induced upregulation of LOX protecting the amount of crosslinks [31] by reducing the impact of blocking some LOX with BAPN [10]. Assuming the mechanism for the effects noted in the previous study was correct, it suggests that the alterations to the magnitude and loading modality of the local mechanical environment were not sufficient to elicit an adequate increase in LOX production that could sustain some BAPN inactivation while keeping active LOX levels high enough to properly crosslink collagen. Because LOX is an extracellular enzyme, diffusion to the posterolateral surface from regions where LOX is upregulated (e.g., the anterior surface) is possible. However, given the diffusivity of similarly sized proteins through the lacunar-canalicular system [32, 33], the time course of the study, and the geometry of the bone, additional LOX on the posterolateral surface due to diffusion from the anterior surface would be minimal.

Structural changes in the form of periosteal expansion were observed due to BAPN in the femur (Table 3) and tibia (Table 4). The effect of BAPN was more noticeable in tibiae because more growth occurs in the tibia over the time course of the study for this age and strain of mouse [34, 35]. The periosteal expansion was likely driven by a qualitatively higher Ps.MAR (Table 2). Although this increase did not reach significance when observed over the last 7 days before sacrifice, a modest increase over the entire 21 day course of BAPN treatment could explain this effect. While it is unlikely that BAPN would stimulate growth in an adult animal, BAPN treatment in a young rapidly growing animal augmented bone modeling to produce a periosteal expansion. Given the skeletal deformities observed in severe cases of osteolathyrism [36], modeling alterations are not unexpected. The confinement of BAPN-induced effects to the cortex is likely caused by the greater relative amount of bone formed between the cortical wall and a trabecular strut over the time course of the intervention [37]. Additionally, the trabecular compartment is more vascularized than cortical bone and the pharmacokinetics of BAPN may be different between these regions giving rise to the disparity in effects.

The treadmill exercise model used here improves the tissue-level mechanical integrity of cortical bone as shown by the increased *K*_c_ in Fig 3. Structural improvements in the trabecular microarchitecture of the femur (Table 5) and tibia (Table 6) were also present. The minimal structural changes observed in cortical bone due to exercise were likely due to higher strains experienced across the knee during running than in the mid-diaphysis and were expected for this sex, strain, and model [20]. No significant changes due to exercise were revealed from 4 point bending of the tibia for either structural- or tissue-level properties (Table 7). The lack of postyield improvements is surprising given the exercise regimen was selected to produce these changes [11, 26]. The lack of significant differences noted here are likely due to the use of female mice in the current study, which may have delayed or mitigated exercise-induced improvements. Female mice were used in an effort to eliminate variable loading environments for each mouse due to cage fighting which can mask the effects of experimental loading and is common in males from this inbred strain [38]. Postyield properties are highly variable even in the most ideal conditions which also likely contributed to the lack of significant differences. Regardless of the lack of differences in 4 point bending, exercise does improve the mechanical integrity of bone tissue. A longer exercise duration and/or a delay between the end of exercise and sacrifice for future studies is recommended [26] to allow the differences in nanoscale indentation modulus and fracture toughness sufficient opportunity to propagate to changes in whole bone bending behavior.

The mild disease state observed here was the principal limitation of the study. BAPN-induced changes to collagen morphology were not accompanied by expected changes in composition or reductions in fracture toughness and ultimate stress [5]. Differences in severity of mechanical effects between the disease state induced here and in other studies are likely due to variations in the dose of BAPN and animal age used. Reduced fracture toughness has been shown at a dose as low as 150 mg/(kg·day) [5] in male mice, but was not observed with the 164 mg/(kg·day) dose used here in female mice. A possible reason why reductions in fracture toughness due to BAPN were not observed in the present study is that at the beginning of the 21 day injection protocol, the mice were 8 weeks of age instead of 5 weeks [5] to ensure the animals were able to complete the exercise protocol. The older age used in the present study still represented rapidly growing animals, but more bone would be produced under the influence of BAPN from age 5 to 8 weeks than from 8 to 11 weeks. There also may be fundamental differences in basal expression or activity levels of LOX between males and females due to estrogen [39, 40]. It is possible to elicit a mechanical response to BAPN using subcutaneous injections in female rodents [41], but this was achieved using more than double the dose used here and administered twice a day. Future studies directly investigating differential effects of BAPN dosage between males and females are recommended. Additionally, while great care was taken to ensure the locations for Raman and AFM were capturing young tissue presumably formed during the BAPN treatment, the fluorescent labels could not be used to select these locations in order to preserve the native hydrated state of bone for Raman and the proper collagen alignment for AFM. The inability to use the fluorescent labels to precisely control for tissue age and select locations formed from days 13 to 20 was a limitation of the study. However, it is very likely that the use of a confocal system to restrict the Raman signal to the outermost layer of bone and the selection of the posterolateral surface for the AFM locations ensured that primarily young tissue formed over the course of the BAPN treatment was interrogated. Additionally, the precise location selection protocol used for AFM was employed so that relative differences between groups were meaningful even if locations included small amounts pre-existing tissue. Because BAPN will only affect newly formed tissue and the expected BAPN-induced changes to collagen morphology are present [10], the methods used here are sufficient to effectively control for tissue age which formed during the treatment period.

While the response to BAPN was less severe than expected, the results reported here and in previous studies indicate that this dose of BAPN did have an effect at the nanoscale over the 8 to 11 weeks of age time frame [10], but it is likely that not enough of the tissue was affected by the BAPN treatment compared to previous studies due to the variations in protocol previously discussed. Future studies investigating the mechanical effects of BAPN should use a higher dose and a longer treatment time in order to ensure a more advanced disease state. While the bulk quantification of crosslinks using high performance liquid chromatography would be inconclusive in the current study due to the modest effect of BAPN and the relative amount of pre-existing tissue to tissue formed during BAPN treatment, quantifying crosslinks in a model where a higher dose was used over a longer period is needed to conclude whether or not the modest effect in this model is due to a failure to adequately inhibit crosslinks or simply enzymatic crosslinks are not as important in determining macroscale mechanical properties as believed. Additionally, the timing of the injections in future studies should be optimized to coincide with the circadian rhythm of bone formation so that over the approximately 2.5 hours when the serum concentration of BAPN is above the half maximal inhibitory concentration of 25 μM bone formation is at its peak [42–45]. Alternatively, other administration routes that do not rely on timing a bolus of BAPN may be more successful in generating an overt mechanical disease state such as osmotic pumps [46] or dietary supplements [47, 48] that deliver near constant or repeated doses over the course of the day to maintain efficacy despite rapid clearance.

## Conclusions

BAPN treatment alters the collagen D-spacing distribution but the shift in D-spacing is not recoverable with concurrent exercise on the posterolateral surface of the femur. While exercise does not affect D-spacing, nanoscale indentation modulus was shifted to higher values with exercise in the absence of BAPN. Exercise-induced improvements in mechanical integrity at the material-level were observed through increased fracture toughness. Future studies should examine the mineral–collagen interface and the contributions of NCPs as a mechanism for exercise-based improvements in fracture toughness observed here and the increased postyield deformation observed in other studies. Because there were no effects on bending behavior for BAPN or exercise, a higher dose of BAPN and longer treatment protocol is recommend for future studies using BAPN and treadmill exercise.
